# Sedation rates of patients with autism spectrum disorder in the emergency department: a case-matched cohort study

**DOI:** 10.3389/fpsyt.2025.1598864

**Published:** 2025-08-12

**Authors:** Ramesh Arasu, Soumitra Das, Naveen Thomas

**Affiliations:** ^1^ Department of Psychiatry, Western Health, Melbourne, VIC, Australia; ^2^ Emergency Mental Health Unit, Western Health – Mental Health and Wellbeing Service, Melbourne, VIC, Australia; ^3^ Department of Psychiatry, University of Melbourne, Melbourne, VIC, Australia

**Keywords:** autism spectrum disorder, sedatives, aggression, emergency department, agitation

## Abstract

**Introduction:**

Adult patients with autism spectrum disorder (ASD) exhibit a range of behaviours that can be disruptive to the medical care of themselves and other patients and as a result, may be at higher risk of requiring chemical sedation. These autistic individuals often experience communication difficulties, sensory sensitivities, and high rates of psychiatric comorbidities, which can exacerbate distress and behavioural dysregulation during acute episodes. This complexity may contribute to an increased reliance on chemical sedation during emergency care. The aim of the present study was to determine whether patients with ASD presenting to hospital for acute psychiatric crises receive more sedative medications compared to similar patients without ASD.

**Methods:**

It is a retrospective case matched control study. 66 presentations from adult patients with a previous diagnosis of ASD who were referred to the mental health team at a single, large emergency department in metropolitan Victoria over the year of 2021 were identified and matched with controls from the same cohort at a 2:1 ratio. The matching was done between age and sex. The number of times each patient was sedated was compared using univariate and multivariate logistic regression, adjusting for other recorded characteristics.

**Results:**

Patients with ASD had 4.83 times the odds of receiving multiple doses of sedatives compared to matched controls when controlling for all non-matched characteristics (95%CI: 1.96-11.94, χ²=64.47, df=10, p<0.001). There was no significant difference in the rates of intramuscular sedation nor mechanical or physical restraint.

**Discussion:**

Clinicians should be aware of the increased rate of receiving multiple doses of sedatives and consider other means of behavioural management in patients with ASD

## 1 Background

### 1.1 Autism spectrum disorder

Patients with Autism Spectrum disorder (ASD) exhibit a range of characteristic behaviours and atypical communication styles that can lead to difficulties establishing relationships with others, and an associated significant degree of psychological agitation (1). Symptom severity is heterogenous. Some patients are completely non-verbal, some require assistance with basic activities of daily living, and others function independently with only mild difficulties (2). Roughly two thirds of patients with ASD have a comorbid psychiatric diagnosis, with the most frequent comorbidities being attention deficit hyperactivity disorder (ADHD), anxiety and depressive disorders or intellectual disability (3–5). These comorbidities complicate the management of these patients, and may further increase their agitation and distress.

### 1.2 Aggressive behaviour

Although it is not a cardinal symptom in the diagnostic criteria for ASD, increased aggression and aggressive behaviours are common manifestations that complicate management. Several retrospective cohort studies suggest that children and adolescents with ASD exhibit aggressive behaviours toward others at a higher rate than the general population, with most measuring rates above 50% (6). Day-to-day aggressive behaviours are less prevalent among adults with ASD, however still increased compared to the general population (7). Adults with ASD also score higher for aggressive factors on subjective rating scales (8). Risk factors for aggression among individuals with ASD include younger age as outlined above, intellectual disability, impaired language and sensory processing (9, 10). This increased propensity for aggression carries a greater need for appropriate behavioural management.

### 1.3 Sedatives

Aggressive and agitated patients are not uncommon in the hospital emergency department. One study in Australia found that security calls for unarmed threats occurred in 3.2 in every 1000 emergency department patients, with the most frequent associated factor being a pre-existing significant psychiatric disorder in over half of patients (11). Another study in 2006 recorded a rate of 5.5 per 1000 emergency presentations (12). These patients should be managed in the first instance by non-pharmacological techniques including verbal de-escalation, the involvement of family and friends and ideally, the movement to favourable environments with more space, fewer neighbouring patients and fewer risky surrounding items. However, in the majority of recorded behavioural agitation events, these methods fail and physical or chemical sedatives are required (13). Chemical sedation is preferred and more frequently used compared to physical restraints due to the significant psychological impact of physical restraints (14). Oral administration is preferred to intramuscular or intravenous sedatives due to easier titration, lower risks of side-effects, and reduced associated psychological distress.

There are significant side-effects associated with modern sedatives, including prolonged fatigue, an increased risk of falls and injuries and addiction (15). Notably, a meta-analytic review found that up to a third of patients who received chemical sedatives in hospital emergency departments experienced adverse events including respiratory depression, QT prolongation and death. Additionally, patients associate a great deal of negative emotion with receiving excessive sedation against their will (16). Benzodiazepines can be associated with a degree of behavioural disinhibition that can last longer than their acute sedative effect. Prolonged courses can lead to tolerance and dependence (17). This article emphasizes that sedatives—both oral and intramuscular—can have disproportionately adverse effects in adults with ASD, particularly when used in settings where trauma-informed or individualized strategies are not employed (18).

### 1.4 Emergency presentations

While patients with ASD remain a relatively rare patient population, their burden on the emergency department should not be underestimated. Children and adolescents with ASD present to the emergency department of hospitals more frequently than those without this diagnosis (19, 20). Adults with ASD also present more frequently for both medical and psychiatric conditions (21). Patients with ASD are more likely to be admitted, and experience longer hospital stays (22, 23). They are more likely to attempt suicide (22, 24). One study found that the healthcare costs to patients with ASD who present to ED were more than double on average compared to other patients without ASD (25). It is therefore important to understand the unique risks and challenges involved with the acute behavioural management of these patients.

Patients with ASD represent a unique and vulnerable patient group that present more frequently to emergency department and are more likely to express aggressive behaviour. This may lead to these patients requiring chemical sedation at higher rates. However, there are no previous studies that have examined the administration of sedatives or use of restraints on patients with ASD in acute settings.

### 1.5 Objectives

Primary: To determine whether adult patients with ASD who present to the emergency department with acute psychiatric illnesses receive more sedatives.

Secondary: To explore the use of intramuscular sedatives, and the use of mechanical or physical restraints.

## 2 Methods

### 2.1 Design, setting and participants

A retrospective case-matched cohort study was conducted involving patients who presented to the emergency department of a major metropolitan hospital (Western Health Emergency Department) in Melbourne, Victoria, Australia, between January 1 and December 31, 2021. The study focused on individuals presenting with acute psychiatric conditions. Patients requiring specialist input were referred to the Emergency Mental Health team, which comprises mental health clinicians and, when necessary, psychiatrists.

The list of patients referred in this way throughout the year of 2021 was manually searched for relevant cases. Participants were required to be over 18 years of age and have a documented formal diagnosis of Autism Spectrum Disorder (ASD). Diagnoses were typically made by a clinical psychologist or psychiatrist using validated assessment instruments such as the ADOS, ADI-R, MIGDAS, or ACIA, in conjunction with a descriptive clinical interview. Researchers verified these diagnostic documents through clinical archives. Those with documentation of an unclear diagnosis, such as those with ‘suspected ASD’ were excluded. ED presentations that were recorded as lasting shorter than 60 minutes were excluded, as these tend not to represent true presentations, but instead show brief presentations where the patient leaves the hospital prior to assessment by any clinician beyond the triage nurse.

A case-matched cohort method was used, where each presentation from a patient with ASD was matched with two control presentations, resulting in a 1:2 case-to-control ratio. Controls had the same sex and an age within three years of associated cases. Controls were manually searched and selected in order of closest proximity to the presenting date of their associated cases. These variables were selected as they were consistently and reliably recorded in the electronic health records and are known to influence behavioural response and sedation thresholds. This manuscript adheres to the STROBE (Strengthening the Reporting of Observational Studies in Epidemiology) guidelines for reporting observational studies.

### 2.2 Data collection

The electronic medical record was then searched for documentation regarding the sedatives that were given during their emergency department presentation. The number of times each patient was sedated was recorded, as well as whether they received intramuscular sedatives or underwent mechanical or physical restraints. It is important to note that, in this ED setting, sedatives (Diazepam, Lorazepam, Haloperidol, Droperidol, Promethazine) are generally ordered by emergency physicians or psychiatrists, based on clinical assessment and in consultation with the mental health team (psychiatrist, senior registrar). The number of doses and route (oral, IM) is determined by patient response, safety risks, and procedural needs. Among patients who received multiple sedative doses, the second dose was typically administered 1–2 hours after the first due to ongoing behavioural risk or agitation. These decisions were clinician-led, not related to procedural requirements, and intravenous or intranasal sedation was not used in any case.

Baseline characteristics were recorded, including a history of alcohol or substance abuse, mood or anxiety disorder, attention deficit hyperactivity disorder (ADHD), personality disorder, psychotic disorder and intellectual disability. Several other ED related characteristics were also recorded, including their reason for presentation, defined as suicide-related or other, their length of stay in ED, and whether police were present during their presentation.

The primary outcome was the administration of multiple sedative doses during the emergency department presentation. Secondary outcomes included the use of any sedatives (versus none), the use of intramuscular sedatives, and the application of mechanical or physical restraints.

### 2.3 Statistical analysis

Data were compiled and stored using Microsoft Excel and analysed using R 4.3.1 (26, 27). Non-matched characteristics were evaluated using Pearson chi-square tests. Univariate and multivariate logistic regression analyses were performed controlling for non-matched characteristics on all primary outcomes using an alpha level of 0.05.

## 3 Results

### 3.1 Matched characteristics

There were 66 presentations from 33 patients with ASD over the year, who were matched with 132 corresponding control presentations. **Table 1** shows the distribution of matched characteristics. There were 43.9% female patients across groups. There was one patient with ASD who had transitioned from female to male, who was matched with male controls. The mean ages across cases and controls were 25.3 and 25.8 respectively.

**Table 1:** Matched characteristics.

Characteristic	Cases (n=66)	Controls (n=132)
Sex*, n (%)*		
Female	29 (43.9%)	58 (43.9%)
Male	37 (56.1%)	74 (56.1%)
Age (years), *mean (SD)*	25.3 (7.2)	25.8 (6.9)

### 3.2 Non-matched characteristics

**Table 2** shows the distributions of non-matched characteristics and associated chi-square results. Patients with ASD were more likely to have ADHD and intellectual disability, while controls were more likely to have psychotic disorders. Patients with ASD were more likely to present due to suicidal ideation or attempt compared to other reasons compared to controls. There were no significant differences in alcohol or illicit drug use, mood or anxiety disorders, cluster B personality disorders, being brought in by police, nor multiple presentations.

**Table 2:** Non-matched characteristics.

Characteristic	Cases (n=66)	Controls (n=132)	χ²	p
History of alcohol or illicit drug abuse, *n (%)*	43 (65.2%)	85 (64.4%)	0.01	0.916
Pre-existing psychiatric diagnoses				
Mood or anxiety disorder, *n (%)*	46 (69.7%)	78 (59.1%)	2.15	0.147
Attention deficit hyperactivity disorder, *n (%)*	22 (33.3%)	6 (4.6%)	28.33	<0.001*
Cluster B personality disorder, *n (%)*	34 (51.5%)	54 (4.1%)	2.00	0.158
Psychotic disorder, *n (%)*	15 (22.7%)	49 (37.1%)	4.32	0.043*
Intellectual disability, *n (%)*	26 (39.4%)	14 (10.7%)	21.48	<0.001*
Presenting complaint				
Suicidal ideation or attempt, *n (%)*	48 (72.7%)	67 (50.8%)	8.99	0.004*
Abnormal behaviour or other, *n (%)*	18 (27.2%)	65 (49.2%)		
Brought in by police, *n (%)*	25 (37.9%)	38 (28.8%)	1.65	0.197
Multiple presentations *n (%)*	44 (66.7%)	71 (53.8%)	3.04	0.085

*Significant p<0.05.

### 3.3 Sedation rates

**Figure 1:**
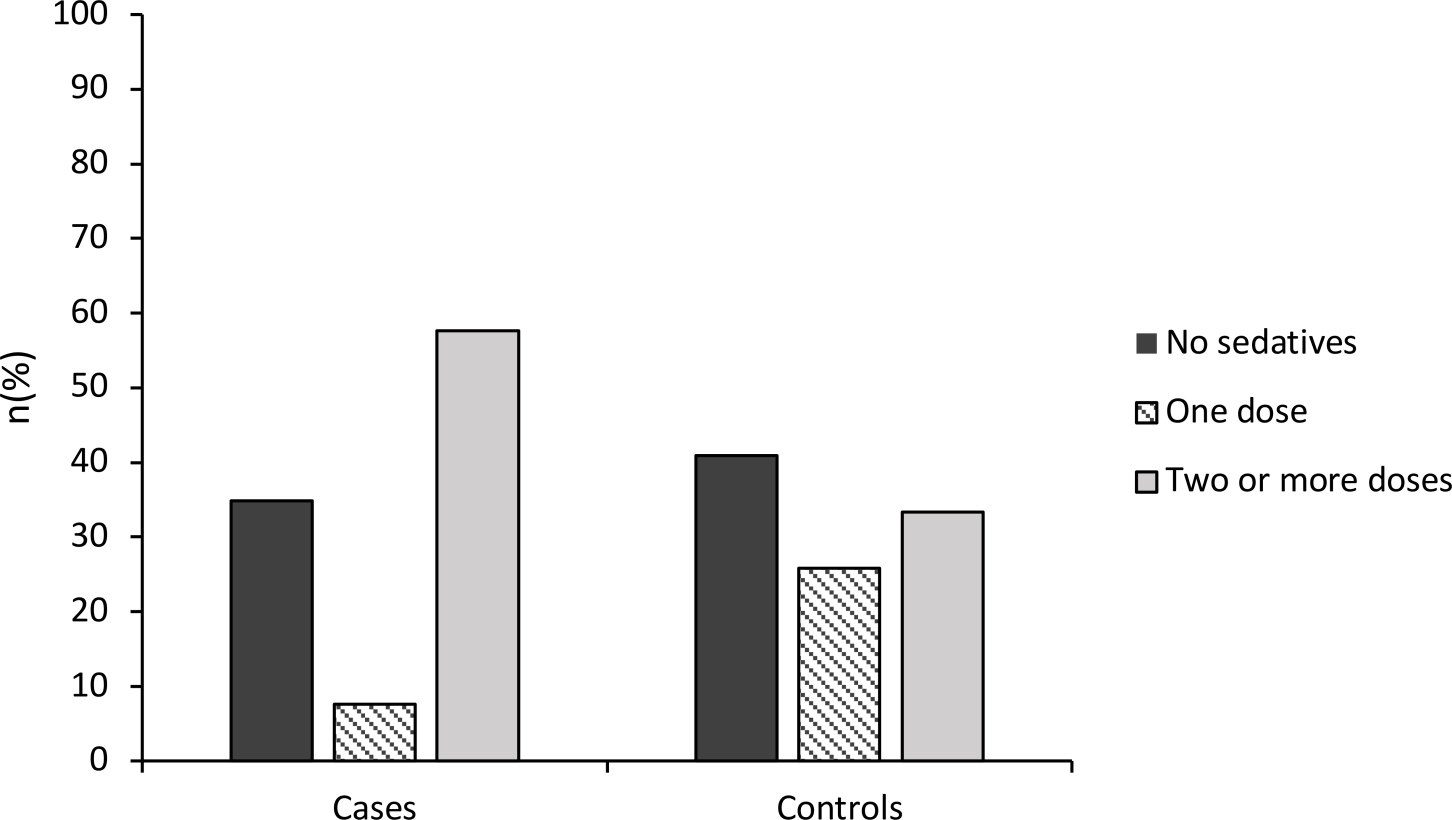
Sedation rates.

A 57.6% of patient presentations with ASD received multiple doses of sedatives compared to 33.3% of controls, as shown in **Figure 1**. Univariate logistic regression was performed to assess this difference, which found that patients with ASD had 2.71 times the odds of receiving two doses of sedatives compared to controls (95%CI: 1.48-4.98), and this difference was statistically significant (χ²=10.60, df=1, p=0.001). Multivariate logistic regression was also performed controlling for all other non-matched characteristics and showed that patients with ASD had 4.83 times the odds of receiving two doses of sedatives compared to controls (95%CI: 1.96-11.94). This finding was statistically significant (χ²=64.47, df=10, p<0.001). There was no significant difference in the rates of receiving any sedatives compared to no sedatives across groups in univariate analysis (χ²= 0.69, df = 1, p = 0.410), and multivariate analysis (χ²= 33.13, df = 10, p = 0.349). These findings are shown in **Table 3**.

**Table 3:** Univariate and multivariate logistic regression.

Comparison (cases vs. controls)	Univariate					Multivariate				
	OR	95%CI	χ²	df	p	OR	95%CI	χ²	df	p
Any sedation	0.77	0.42-1.43	0.69	1	0.410	0.67	0.29-1.54	33.13	10	0.349
Multiple sedative doses	2.71	1.48-4.98	10.60	1	0.001*	4.83	1.96-11.94	64.47	10	<0.001*
Intramuscular sedatives	1.68	0.64-4.43	1.18	1	0.294	1.26	0.39-4.10	37.76	10	0.703
Mechanical or physical restraint	1.53	0.64-3.63	0.97	1	0.336	1.75	0.54-5.61	45.11	10	0.350

*Significant p<0.05.

### 3.4 Intramuscular sedation rates

**Figure 2:**
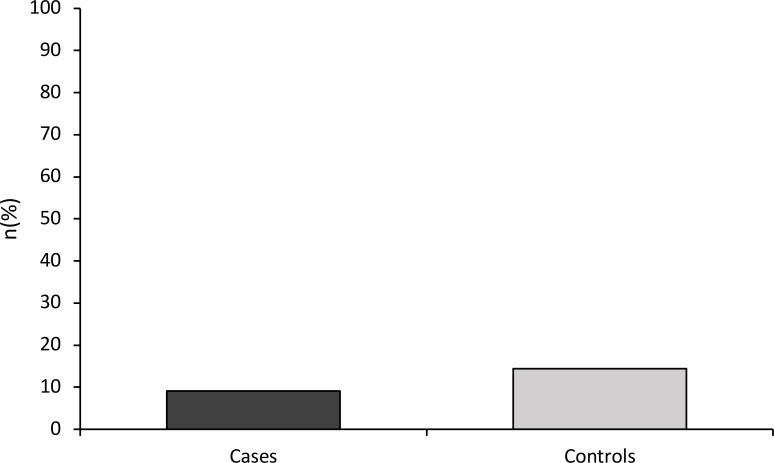
Intramuscular sedation rates.

A 9.1% of patient presentations with ASD received intramuscular sedatives compared to 14.4% of controls, as shown by **Figure 2**. However, this difference was not significant in univariate analysis (χ²= 1.18, df = 1, p = 0.294), and multivariate analysis (χ²= 37.76, df = 10, p = 0.703). The results of these analyses are shown in **Table 3**.

### 3.5 Restraint rates

**Figure 3:**
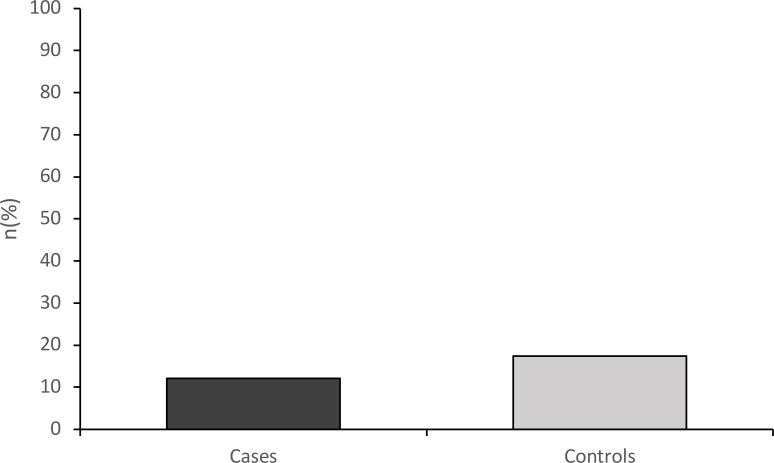
Mechanical or physical restraint rates.

A 12.1% of patient presentations with ASD underwent physical or mechanical restraint, compared to 17.4% of controls, as shown by **Figure 3**. However, there was no significant difference in rates of restraint in univariate analysis (χ²=0.97, df = 1, p = 0.336), and multivariate analysis (χ²=45.11, df = 10, p = 0.350). The results of these analyses are shown in **Table 3**.

## 4 Discussion

### 4.1 Sedatives

This study suggests that patients with ASD have higher odd ratios of receiving multiple doses of sedatives in the emergency department compared to patients who present similarly but do not have a prior diagnosis of ASD. This represents a potentially increased degree of harm to from prolonged sedative effects and psychological distress. Increased sedation may be necessary for patients with ASD, as these patients are more likely to express aggressive behaviours toward others and injurious behaviours toward themselves (28). On the other hand, it may also be possible that physicians have a lower threshold to use sedatives because they fail to recognise the unique difficulties with communication and behaviour exhibited by these patients. A primary presenting complaint of a psychiatric nature increases the risk of receiving sedation or restraints in the emergency department (13). However, it is unclear whether treating physicians have a lower threshold to sedate such patients, or whether such patients truly require sedation over less invasive means of behavioural management.

### 4.2 Intramuscular sedatives and restraint

While there is reason to believe patients with ASD are at higher risk of receiving intramuscular sedatives and mechanical or physical restraints, there was no significant difference in rate observed in the present study. This contrasts with findings from previous studies in paediatric populations, where children with ASD and ADHD were more likely to be restrained or secluded compared to their neurotypical peers. The differing pattern in our study may reflect differences in the adult versus paediatric ASD populations, including developmental stage, service environment, and behavioural adaptation over time (29). Our finding is reassuring, as provides evidence that there is no such increased risk to patients with ASD. However it should be noted that intramuscular sedation is rarely used for all patients and it is possible that the present study was underpowered to detect this difference. Future studies would require larger sample sizes to further explore these risks.

### 4.3 Limitations

The major limitation of this study was small sample size and associated statistical power. Although patients with ASD with acute psychiatric conditions are overrepresented in the emergency department compared to the general population, they are still very small in overall number (20). Given the small size of the ASD cohort, multiple presentations were included to maintain statistical power. While this may introduce bias due to non-independence of observations, matching was done with age- and sex-matched controls, and multivariate models adjusted for key clinical differences. A case-matched cohort design was used along with dichotomous outcome variables to overcome this limitation, however we still failed to observe a significant difference for secondary outcomes.

### 4.4 Non-pharmacological interventions

Children with ASD are more likely to exhibit disruptive behaviours including physical and verbal aggression toward others and self-injury (30). There are a significant number of interventions aimed at young children with ASD, which have been studied extensively (31, 32). However, there is a lack of published literature focusing on adults with ASD. Prior research for adults has focused on long-term medications and functional assessments rather than behavioural interventions or acute de-escalation techniques (33). Longitudinal studies suggest that challenging behaviours vary widely in adult patients with ASD, and suggest that these behaviours may decrease as patients get older (34). They are also more likely to have received behavioural therapies and psychological interventions, which may attenuate their symptoms. Such patients may exhibit the characteristic speech patterns and communication difficulties associated with ASD, but may also be more competent at controlling their aggressive and disruptive behaviour (35). Further research into acute interventions for adult patients with ASD who are experiencing acute psychological distress may assist in reducing the need for sedation and potential associated risks.

## 5 Conclusion

Attempts should always be made to reduce the use of sedative medications in favour of other techniques for behavioural management. These include verbal de-escalation, reducing sensory stimuli, one-to-one nursing, prompt security presence and the involvement of family or friends. Since patients with ASD are at a higher risk of receiving multiple sedatives, efforts should be made to recognise their patterns of behaviour and difficulty, to understand them and formulate constructive and safe ways to manage their behaviour.

## Data Availability

The original contributions presented in the study are included in the article/supplementary material. Further inquiries can be directed to the corresponding author.
